# The RNA-seq based endometrial receptivity test (rsERT) compared to pinopode: A better diagnostic tool for endometrial receptivity for patients with recurrent implantation failure in Chinese population

**DOI:** 10.3389/fendo.2022.1009161

**Published:** 2022-10-21

**Authors:** Jingjing Chen, Aihua He, Qiong Zhang, Jing Zhao, Jing Fu, Hui Li, Yanping Li

**Affiliations:** ^1^ Department of Reproductive Medicine, Xiangya Hospital, Central South University, Changsha, China; ^2^ Clinical Research Center for Women’s Reproductive Health in Hunan Province, Changsha, China

**Keywords:** endometrial receptivity, window of implantation, pinopode, gene profiling, transcriptomic (RNA-Seq), rsERT, personalized embryo transfer

## Abstract

Displaced window of implantation (WOI) is one of the endometrial origins that accounts for implantation failure, especially for patients with recurrent implantation failure (RIF), yet no standard diagnostic tool has been recognized. The study consists of two parts, aiming to compare the concordance and efficacy of the diagnostic tools, the newly developed RNA-seq based endometrial receptivity test (rsERT) to the conventional pinopode, in diagnosing WOI and guiding personalized embryo transfer (pET). With the same group of RIF patients, the rsERT diagnosed 32 patients (65.31%) with normal WOIs, and most of the displacements were advancements (30.61%). While according to pinopode, only 14 patients (28.57%) were found with normal WOIs, and most patients (63.27%) presented delayed growth patterns. After conducting pET, patients in the rsERT group had higher successful pregnancy rates while requiring fewer ET cycles (50.00% vs. 16.67%, p=0.001). The study proved poor consistency between the diagnostic tools of endometrial receptivity based on cellular structure and gene profiling, and it supported rsERT as a reliable tool with potential clinical value.

## Background

Successful embryo implantation is a crucial event that relies upon elaborate communication between the capable embryo and the receptive endometrium (1). Although there have been significant advances in embryo culture technology, endometrial receptivity remains a hurdle in today’s ART (2). In humans, the endometrium undergoes dynamic and complex changes during the menstrual cycle and becomes receptive to blastocysts in a brief period during the mid-luteal phase, which is known as the window of implantation (WOI), usually around days 19-23 of the menstrual cycle (3). However, WOIs could be variant among patients, and implantation failure may occur despite a viable embryo due to embryo-endometrium asynchrony (4, 5). It has been suggested that the implantation failure of endometrial origin is not a result of pathology but a failure to synchronize the developing embryo with a receptive endometrium (6), thus making diagnosing WOI a critical task, especially for patients with repeated implantation failure(RIF).

Pinopode has been considered one of the standard indicators that have been proposed and extensively studied with WOI and fertility. Pinopodes are smooth mushroom or balloon-shaped projections that arise from the apical surface of the luminal epithelium of the endometrium in mice, rats, and humans (7). Its development coincides with the implantation window, as well as other receptivity changes including loss of progesterone receptors (8), peak expression of integrins (9), osteopontin (10), LIF (11), etc., indicating a possible function in embryo implantation. However, strong disagreement still exists in the literature as to the function and clinical value of pinopode, and the necessity of pinopodes in the implantation process has yet to be firmly established (12). Using pinopode as an indicator of endometrial receptivity was considered a promising method. Poor IVF outcomes were related to altered pinopode shape and poor pinopode development (13–17), and personalized embryo transfer(pET) based on pinopode scoring has been proved feasible (15, 18). However, other investigators held uncertain attitudes. Some found pinopode presented throughout the luteal phase rather than a specific period during WOI, and women experiencing infertility do not exhibit a significant difference in pinopode coverage or morphology (19–21).

Recent developments in omics have heightened the need for personalized medicine, and earlier studies proved the feasibility of distinguishing different phases of the menstrual cycle or different diseases based on transcriptomic characteristics (22–24). The disrupted gene expression pattern was found in RIF patients, and 12.0% ~59.2% of RIF patients were found with displaced WOIs, significantly higher rates compared to the controls (25–30). Personalized embryo transfer based on transcriptomic tools has been proven feasible and valuable, especially for RIF patients. Significantly higher pregnancy rates were reported in the pET group instructed by the commercial Endometrial Receptivity Array (ERA) test, and rescue of non-receptive patients by pET in a displaced WOI resulted in similar clinical outcomes to controls (25, 31–34). Other investigators, however, considered the value was rather limited when applied ERA in ICSI cycles, first embryo transfer cycles, and in good prognosis patients considering the current level of development (26, 30, 35–38). Based on RNA-seq technology while aiming to improve the diagnostic tool, especially for Chinese population, we constructed an RNA-seq based endometrial receptivity test (rsERT) in a previous study and validated its clinical value in RIF patients (29). Prospective clinical trials, including multicenter trials, are being conducted for further validation.

Previous studies have demonstrated poor concordance between the ERA tool and traditional histologic dating while supporting the superiority of the transcriptomic tool in diagnosing WOI-displacement (39, 40). However, a comparison between transcriptomic tools and pinopode evaluation is yet to be reported. In this study, we aim to compare the concordance and efficacy of the tools, the pinopode and rsERT, in diagnosing WOI and instructing personalized embryo transfer.

## Materials and methods

### Study design and patients

**Figure 1:**
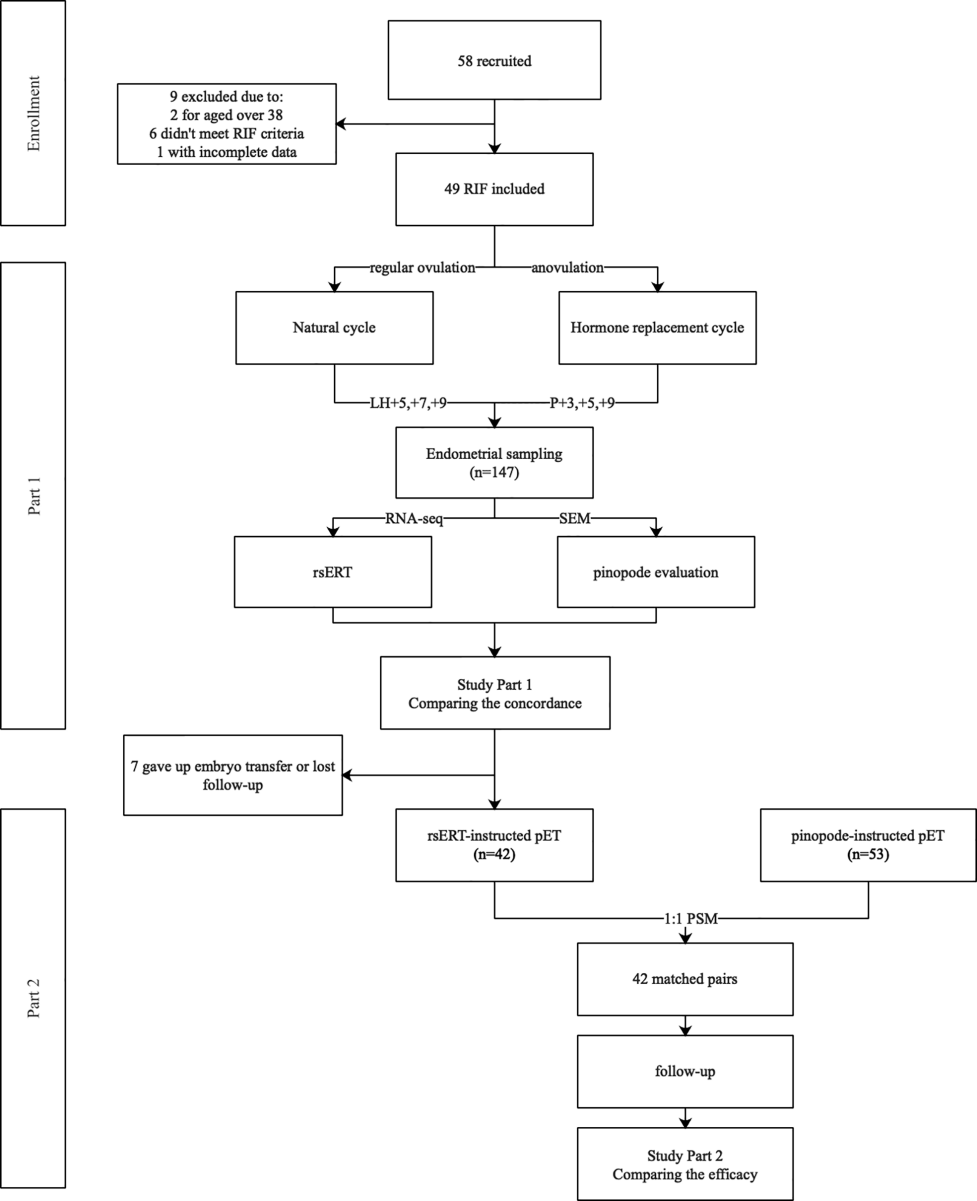
Flow chart of the study. A total of 58 RIF patients were recruited in Part 1 of the study, 9 were removed due to variant of reasons. Three consecutive endometrial biopsies from a same menstrual cycle were obtained from each patient, being sent for both rsERT test and pinopode evaluation, and the concordance was evaluated. In Part 2, 42 patients that received rsERT instructed pET were matched with 42 patients who once received pinopode-instructed pET in our center by using PSM in a 1:1 ratio. Clinical outcomes were followed up to compare the efficacy of the diagnostic tools.

This study was conducted at the Department of Reproductive Medicine of Xiangya Hospital, Changsha, Hunan, China from November 2017 to July 2019 and consists of two separate parts (**Figure 1**). Study Part 1 aims to compare the concordance of the diagnostic tools of endometrial receptivity, the rsERT and pinopode, in a paired biopsies population, in which endometrial biopsies obtained from the same patient were divided and sent for both tests. The study was approved by the ethics committee of the department of reproductive medicine, Xiangya Hospital, Central South University (reference number 2017002) and registered in the Chinese Clinical Trial Registry (http://www.chictr.org.cn/.Registration No. ChiCTR-DDD-17013375). Part 2 aims to compare the accuracy and efficacy of the tools in instructing pET. The pregnancy outcomes of RIF patients with rsERT- or pinopode- instructed pET cycles were compared retrospectively. The study was approved by the same ethics committee (reference number 2019016). Patients were informed and consented to the use of their anonymized data for research purposes. Informed consents were obtained from all the patients.

### Inclusion and exclusion criteria

The inclusion criteria included age between 20 and 38, a body mass index (BMI) of 18-25 kg/m^2^, and a history of RIF, which was defined as failure to achieve a clinical pregnancy after receiving at least 4 morphologically high-quality cleavage embryos or 2 high-quality blastocysts in a minimum of 2 fresh or frozen transfer cycles. Patients were excluded for the following reasons: endometrial lesions (such as intrauterine adhesions, endometrial tuberculosis, endometritis, endometrial hyperplasia, and a thin endometrium, etc.); severe hydrosalpinx; severe endometriosis (stage III-IV); uterine malformations; other medical complications (including hypertension, diabetes, etc.). The criteria for embryos of high quality were as follows: (i) cleavage-stage embryo: 20% fragmentation or less on day 3 and absence of multinucleated blastomeres (41); (ii) Blastocysts: ≥3BB on day 5 and 6 (42).

### Endometrial preparing and sampling

In Part 1 of the study, three endometrial biopsies were taken consecutively during the same menstrual cycle, while avoiding repeated sampling from the same uterine wall. 49 RIF patients consented to participate in the study and 147 endometrial biopsies were collected. All procedures were performed following the applicable rules and guidelines. All patients were informed and written consents were obtained.

For endometrial preparation, patients with or without regular ovulation used ovulation monitoring (natural cycle) and hormone replacement therapy (HRT), respectively. In brief, for ovulatory patients, ultrasound monitoring was initiated from Day 10 of the menstrual cycle and plasma LH was dynamically measured when the diameter of the dominant follicle was ≥14mm. The LH peak day was recorded as LH+0 and endometrial biopsies were obtained on 5, 7 and 9 days thereafter (LH+5/+7/+9) using an endometrial sampler (AiMu Medical Science & Technology Co.; Liaoning; China). For anovulatory patients, hormone replacement treatment (HRT) was applied. Estradiol administration was started on Day 3 of the menstrual cycle, and progesterone was added after at least 12 days if the endometrium was >7 mm. The first day with progesterone supplementation was seen as P+0, and endometrial tissues were obtained after 3, 5 and 7 days (P+3/+5/+7).

Specimens were first rinsed in saline and then divided evenly. One was stored in RNA-later buffer (AM7020; Thermo Fisher Scientific, Waltham, MA, USA) for RNA sequencing and rsERT testing, while the other was fixed in 2.5% glutaraldehyde solution for more than 48 hours, rinsed twice with PBS buffer, and dehydrated in series of ethanol concentrations (50%, 70%, 80%, 95%, 100%). The samples were then dried in a critical point drier using carbon dioxide and coated with palladium gold before being sent for scanning electron microscopy (SEM) and pinopode evaluation.

### WOI delineation

In humans, pinopode morphology changes during the luteal phase. Three different pinopode development stages have been identified, known as developing, fully developed, and regressing, with each phase lasting approximately 24 hours (43, 44). In brief, developing pinopodes are smooth and slender membrane projections that arise from the entire cell apex; fully developed pinopodes are maximally folded, smooth and devoid of microvilli; and regressing pinopodes are slightly wrinkled and microvilli tips reappear. In the study, 10 fields at a magnification of 2,000 were randomly chosen under the SEM, and two independent observers were responsible for evaluating pinopode. By observing endometrial biopsies, a consecutive growth pattern of pinopode of a patient was obtained, and the most receptive day was determined to be the day on which the most fully developed pinopodes appeared, the day after developing pinopodes appeared, or the day before regressing pinopodes appeared.

For rsERT testing, the tool comprises 175 biomarkers genes and demonstrated an average accuracy of 98.4% *via* tenfold cross-validation. It can distinguish precisely between pre-receptive, receptive, and post-receptive endometrium while remaining unaffected by different endometrium preparing methods (HRT or natural) (29).

### Personalized embryo transfer and propensity score matching

Part 2 of the study aims to compare the efficacy of the tools in instructing personalized embryo transfer (pET). rsERT group comprises 42 patients from Part 1, who consented to receive rsERT-instructed embryo transfer. Additional 53 patients who had previously received pinopode-instructed personalized embryo transfer were retrospectively included and compared. Propensity score matching (PSM) was used considering the potential bias introduced by the retrospective design. Groups were matched for age, BMI, endometrium preparation method and number of transferred high-quality embryos in a 1:1 ratio. After matching, 42 pairs of patients were selected for statistical comparisons out of a total of 95.

For both examination and transfer cycles, the same endometrial preparing method was used. One or two embryos were transferred, aiming to synchronize blastocysts with the predicted optimal WOI. Day 3 cleavage-stage embryos were transferred 2 days earlier accordingly.

The primary outcome was intrauterine pregnancy rate (IPR), which was defined as the presence of a gestational sac containing a fetal heart as evaluated by ultrasound. Secondary outcomes were the live birth rate (LBR) and implantation rate (IR). LBR refers to the number of pET cycles that result in deliveries. IR refers to the number of gestational sacs observed divided by the number of transferred embryos.

### Statistical analysis

SPSS Statistics 25.0 was used for statistical analysis. Chi-square test, independent sample t-test, Mann Whitney-U test and Cohen’s Kappa index were used for statistical description and comparison. P-value <0.05 was considered statistically significant.

## Results

**Figure 2:**
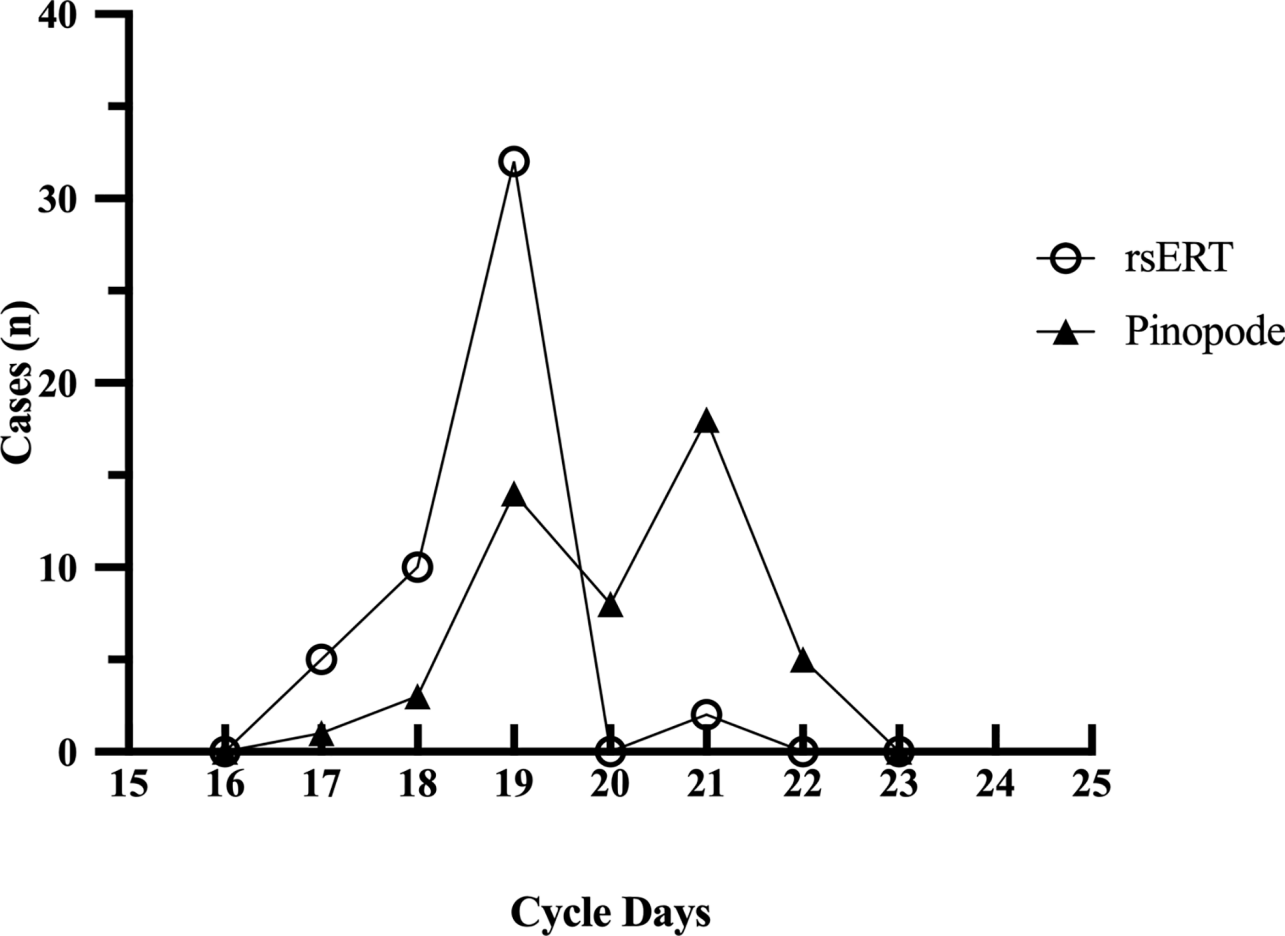
Optimal WOIs predicted by rsERT and pinopode of a same RIF group (n=49). The rsERT diagnosed 65.31% of the patients (n=32) with normal WOIs (including 2 patients with prolonged-WOIs), and most of the displacements were advancements (15/49, 30.61%). While according to pinopode, 28.57% (n=14) of the same group were considered with normal-WOIs and 63.27% (n=31) presented delayed pinopode growth pattern.

In the first part of the study, 147 endometrial biopsies were collected from 49 RIF patients and sent for both tests. The demographic characteristics of patients are detailed in **Supplementary Table S1**. According to rsERT, the majority of patients (32 of 49, 65.31%) had normal-WOIs (on P+5 or LH+7), while two of them were found with prolonged-WOIs, that their endometrium remained receptive for more than 72 hours (**Figure 2**). The remaining 17 patients were diagnosed with displaced-WOIs, of which 15 were advanced (30.61%) and 2 were delayed (4.08%). In contrast, by evaluating pinopode, only 14 patients (28.57%) were considered to have normal-WOIs. Most patients (31 of 49, 63.27%) presented delayed pinopode growth patterns and four patients (8.16%) were diagnosed with advanced WOIs. Test results for only 15 patients (30.61%) were congruent, indicating poor consistency.

42 of the 49 participants consented to receive personalized frozen embryo transfer following the instruction of the rsERT. There is no intervention for the 2 patients who were found to have prolonged-WOIs. 23 patients (54.76%) conceived successfully after transferring one or two embryos to match the predicted optimal WOI. Their basic information and WOI distributions were presented in **Table 1** and **Supplementary Table S2**. While WOIs were confirmed by pregnancy, only 8 patients (34.78%) exhibited concordant pinopode patterns, with a Cohen’s Kappa index of 0.090 (P=0.112), demonstrating poor concordance.

**Table 1:** Basic information and predicted optimal WOIs of the 23 pregnant patients.

No.	Age(y)	Infertility Type^1^	Previous Failed Cycles(n)	No. of Previous Transferred good-quality embryos(n)	rsERT^2^	Pinopode	pET Cycles(n)
**1**	31	1	3	4	N	D	1
**2**	27	1	3	3	N	D	1
**3**	32	2	3	5	N	N	1
**4**	31	1	4	8	A	D	1
**5**	32	2	7	10	N	D	1
**6**	30	1	4	8	A	D	1
**7**	29	1	2	1	D	N	1
**8**	34	1	2	3	N*	N	1
**9**	33	2	3	4	D	D	1
**10**	31	2	2	3	N	D	1
**11**	36	4	2	3	N	N	1
**12**	27	1	2	4	N	D	1
**13**	28	1	2	3	A	D	1
**14**	34	1	4	6	N	N	1
**15**	34	2	2	4	N	D	1
**16**	33	1	4	5	A	D	1
**17**	28	1	2	3	A	D	1
**18**	35	1	6	3	N	N	1
**19**	32	2	2	2	N	D	2
**20**	27	1	4	6	N	N	2
**21**	32	1	2	2	A	A	2
**22**	31	1	3	3	N	D	2
**23**	37	1	3	4	N	D	3

Basic information and predicted optimal WOIs of the 23 patients who conceived successfully after rsERT-instructed pET. ^1^Infertility type:1= primary infertility, 2 = secondary infertility; ^2^Predicted optimal WOI by rsERT or pinopode evaluation: A = Advanced WOI (P+3/+4 or LH+5/+6), N = Normal WOI (P+5 or LH+7), D = Delayed WOI (P+6/+7 or LH+8/+9). ^*^Patient No.8 was diagnosed with prolonged-WOI by rsERT, from P+5 to P+7.

Notably, there are 7 patients who once received pinopode-instructed pET in our center but failed to conceive after several attempts. They were recruited for the rsERT test, and 6 of them received a different result. Five patients conceived on the first attempt after pET (**Supplementary Table S3**).

To evaluate the accuracy and efficacy of the tools, patients with rsERT- or pinopode-instructed pET histories were included retrospectively, and their clinical outcomes during the first pET cycle were compared as shown in **Table 2**. 42 pairs of patients were matched for age, BMI, endometrial preparation method, and number of transferred high-quality embryos with PSM in a 1:1 ratio. Age, BMI, infertility type, baseline FSH, LH, AMH and number of previous transferred cycles were comparable between groups; however, but infertility duration of patients in rsERT group was significantly longer. Particularly, a higher proportion of patients were diagnosed with displaced-WOI in the pinopode group.

**Table 2:** Baseline information and clinical outcomes of patients that received pET (after PSM).

Parameter	rsERT	Pinopode	P-value
**Patients, n**	42	42	–
**Age, y**	31.93 ± 3.07	31.56 ± 3.30	0.636
**BMI, kg/m^2^**	21.10 ± 2.23	21.30 ± 2.49	0.719
**Infertility duration, y**	5.74 ± 3.61	4.05 ± 2.61	**0.019**
**Infertility type**			
**Primary infertility**	28	22	0.182
**Secondary infertility**	14	20	
**Etiology of infertility**			
Tubal	33	39	0.176
Endometriosis	1	1	
DOR	5	2	
Other	3	0	
**Previous transferred cycles, n**	3 (2-4)	3 (2-3)	0.134
**Baseline FSH, mIU/ml**	6.82 (5.85-8.15)	6.10 (5.10-7.15)	0.074
**Baseline LH, mIU/ml**	4.96 (4.19-6.47)	4.90 (4.01-6.83)	0.687
**AMH, ng/ml**	3.454 ± 3.576	2.594 ± 1.512	0.431
**Displaced-WOI, n (%)**	12 (28.57)	34(80.95)	**0.000**
**Endometrial preparing method, n**			
HRT	19	27	0.079
NC	23	15	
**Endometrial thickness, mm**	9.10 (7.90-10.90)	9.10 (8.00-10.70)	0.707
Endometrial pattern*, n			0.602
A	8	7	
B	31	34	
C	3	1	
**P levels on the day of progesterone administration**	0.11(0.08-0.30)	0.30(0.24-0.40)	0.056
**No. of transferred embryos, n**	75	73	0.590
**Stage of embryo development, n**			
D3	36	41	0.320
D5	39	32	
**No. of good-quality embryos, n**	52	64	**0.007**
**Intrauterine pregnancy rate, n (%)**	21(50.00)	7(16.67)	**0.001**
**Implantation rate, n (%)**	25(33.33)	8(10.96)	**0.001**
**Miscarriage rate, n (%)**	3(14.29)	1(14.29)	0.708
**Ectopic pregnancy rate, n (%)**	1(2.38)	0(0.00)	0.500
**Live birth rate, n (%)**	18(42.86)	6(14.29)	**0.004**

Values presented as mean ± SD or median (IQR). Endometrial thickness and endometrial pattern were measured on the day of progesterone administration. *Endometrial pattern was classified as A (a triple-line pattern), B (an intermediate isoechogenic pattern) and pettern C (homogenous hyperechogenic pattern). Bold value represents statistically significant.

All the patients received pET instructed either by rsERT or pinopode. No statistically significant differences were observed between the groups in terms of the endometrial preparation method, endometrial thickness, or endometrial pattern. Although more high-quality embryos were transferred in the pinopode group, the intrauterine pregnancy rate, implantation rate and live birth rate were significantly higher in the rsERT group (50.00 vs. 16.67%, P=0.001; 33.33% vs. 10.96%, P=0.001; 42.86% vs. 14.29, P=0.004). Three patients in the rsERT group and one patient in the pinopode group had miscarriages due to embryonic chromosomal abnormalities and fetal malformation. One patient experienced an ectopic pregnancy.

**Figure 3:**
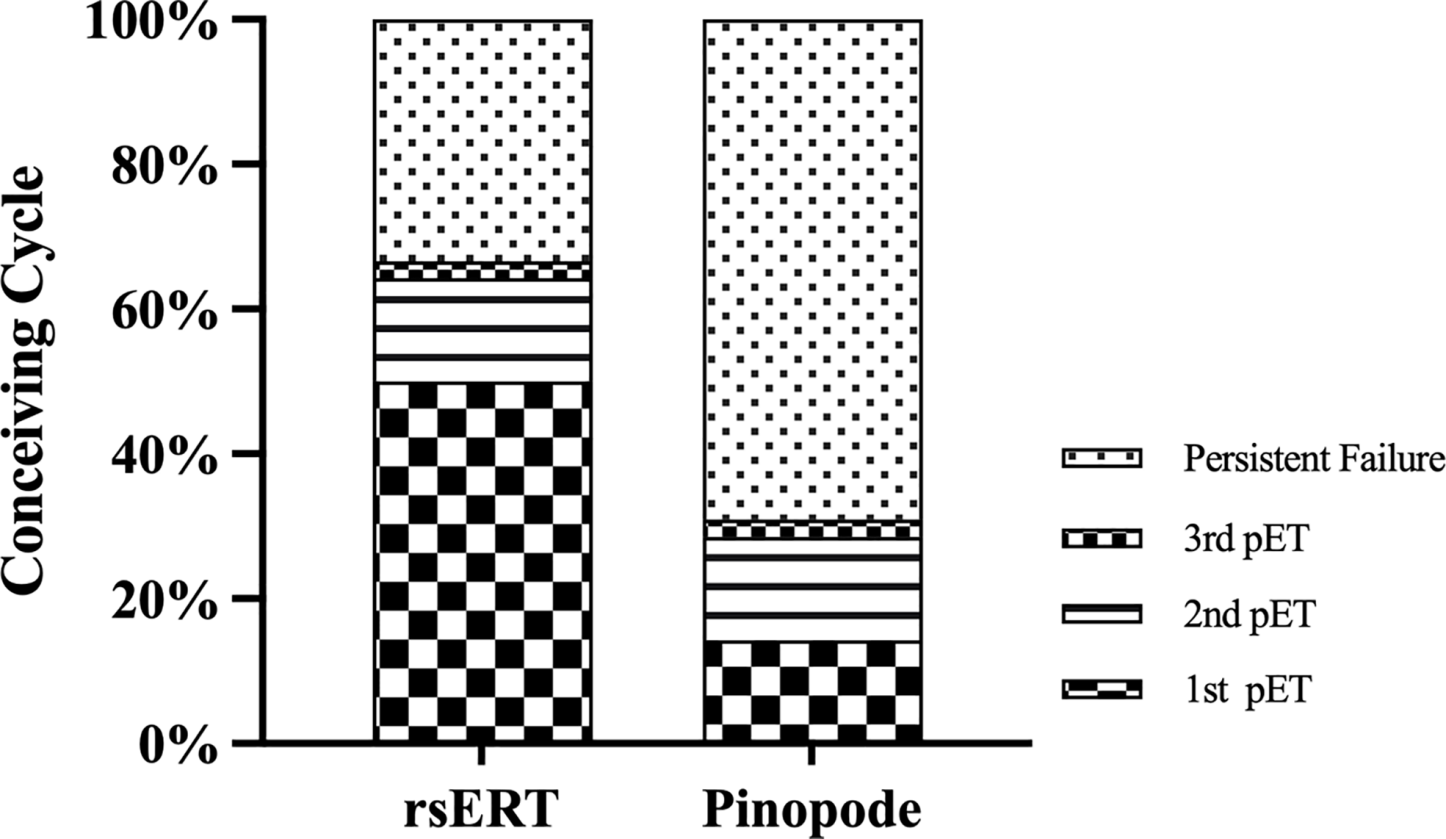
Histogram with the distribution of conceiving cycle between the groups. Patients in the rsERT group have a higher successful pregnancy rate and require fewer ET cycles.

Patients in the rsERT group have a higher successful pregnancy rate while requiring fewer ET cycles, as shown in **Figure 3**. 21 of 42 patients conceived successfully in the first ET cycle compared to 7 of 42 of the pinopode group (50.00% vs. 16.67%, P=0.001). 14 and 29 patients, respectively, remained persistent infertile after three attempted treatment cycles (33.33% vs. 69.05%, P=0.001).

## Discussion

Endometrial receptivity is a crucial event that its understanding has been one of the centers of dispute for researchers studying human reproduction (45). While histological and morphological markers being gold standards for almost half of a century, their accuracy and consistency are contested (46, 47). Rapid developments took place in omics technology in the past few decades from theoretical research to clinical applications, providing us with novel insights and a deeper understanding of endometrial receptivity (28). However, clinical evidence is required before widespread use.

Previous studies found poor consistency between the transcriptomic tool and histological dating while supporting the superiority of the former (38–40), here we bridge the gap by presenting the first evidence comparing rsERT to pinopode evaluation in predicting WOI and instructing personalized embryo transfer, where similar poor concordance was found. According to rsERT, most RIF patients had normal WOIs, and the majority of displaced WOIs were advanced, which is consistent with earlier research (30, 31, 38, 40). However, most of the same group present delayed pinopode growth patterns.

Pinopode has been suggested as a controversial marker of endometrial receptivity. It displays cycle-dependent changes in morphology and is most prominent during the putative WOI (48). Several studies established pinopode as a reliable indicator of WOI, and pinopode-instructed pET showed promising clinical outcomes. However, its indicator role in humans remains obscure and controversial (12). Prior studies found no significant difference in pinopode morphology and coverage between the recurrent pregnancy loss group and the typical fertile group (20) or between infertile patients with and without endometriosis (49). According to our previous work, patients presenting with few or no mature pinopode nevertheless have a chance of becoming pregnant (15). Possible explanations include the randomness of sampling, the limited fields being counted, and the inevitable subjectivity and variability between observers. Endometrium luminal surface is highly heterogeneous. It is found that most of the endometrial samples obtained from the luteal phase showed only 5% to 20% coverage of pinopodes (19). While increasing the number of counting fields may help reduce sample error, as recommended 60 fields in each specimen for calculating pinopode coverage (13), it is impractical for clinical use compared to other diagnostic tools such as ultrasound, histological analyze, ERA or rsERT, etc. Another suspicion was raised by Usadi et al., who found pinopodes persisted for the entire duration of the secretory phase (48), accordant with Quinn et al., who reported pinopodes present throughout the luteal phase of the menstrual cycle, even up to the eleventh week of pregnancy (19), contradicting the perceptive role of an indicator.

The transcriptomic tools demonstrate natural advantages in terms of objectivity and precision. According to rsERT, the transcriptomic signatures of endometrium were identified and classified into pre-receptive, receptive, and post-receptive stages, hence establishing a precise individualized WOI and aiding clinical decisions. According to our previous study, pET based on pinopode could assist 33.82% of RIF patients to achieve clinical pregnancy, whereas only 8.11% of patients in the conventional FET group conceived (15). Here by conducting rsERT-instructed embryo transfer, both the intrauterine pregnancy rate and the implantation rate were further elevated (50.00% vs. 16.67%, 33.33% vs. 10.96%), and patients in the rsERT group require fewer ET cycles for a successful pregnancy. Similar promising results were observed by applying pET based on ERA, the cumulative pregnancy rate was significantly higher in the pET group compared with the regular ET group (93.6% vs. 79.9%) (33), and a comparable pregnancy rate was obtained in the receptive patients and nonreceptive patients (58.8% vs. 50.0%) (31). All of which demonstrated the feasibility and potential value of the tools.

In this study, we found 65.31% of RIF patients with normal WOIs, however, for patients that received pET, 33.33% of them remained persistent failure after multiple attempts. That, apart from a displaced-WOI, other factors coexisted still hampering successful pregnancy. Disrupted WOI might be one of the possible explanations. By analyzing endometrial transcriptomics of RIF patients, Koot et al. (28) considered RIF to be a result combining displacement and disrupted WOI, whereas Leon et al. (50) suggested that those factors either alone or together lead to repeated implantation failure. Other factors, including hyperplasia, submucosal myomas, endometrial polyps, endometritis, etc., are common reasons hampering embryo implantation (51). However, despite all the pathologies being corrected, insufficient endometrial receptivity might persist. While modified embryo transfers could rescue asynchrony, pathology remains a tough problem.

Another notable issue is the between-cycle consistency of the tools. Considering its invasive nature and the expected indicating role, most of the tests are conducted before the transfer cycle, making cycle variability an inescapable question. As for pinopode, a study conducted by Ordi et al. shows that, when taking one biopsy per cycle for three consecutive spontaneous cycles, most of the patients showed poor intra-patient consistency in pinopode scores that indicated low predictive value (52). While considering transcriptomic tools, a previous study recruited seven women for a second ERA test on the same day, approximately one month later, where consistent results were found (39). However, Arianne et al. (40) found poor reproducibility of the ERA test, as only 5 of 14 patients initially diagnosed as “non-receptive” with ERA obtained the same diagnosis. Further studies with larger populations are needed before a safe conclusion.

While transcriptomics revolutionized the field, its expense and invasive nature continue to be obstacles. Displaced-WOIs were identified in 24% to 84.9% of RIF patients and 12% of the controls (25, 26, 30), and rescue of “non-receptive” was found a promising therapy (25, 29, 31). However, it has been shown that ERA does not improve pregnancy outcomes compared to standard ET strategy in the first embryo transfer cycles, or in populations including RIF or patients with good prognosis (26, 36, 38). Considering the present state of research, transcriptomic tools were not recommended as a routine test for unselected patient populations (26). As it has been reported, half of the couples quit before any fertility treatment was started, and one-third stopped after at least one IVF cycle (53). Psychological stress and lost hope for success are the primary considerations. Therefore, while diagnosing recurrent implantation failure calls for consideration and prudence, for patients who have experienced two or more failed cycles, positive actions, including endometrial receptivity assessments, are recommended.

Lastly, our study has points of limitations. Firstly, we took three endometrial samples from the same patient at 48-hour intervals to observe a growth pattern of pinopode. Although we attempted to avoid repeated sampling from the same uterine wall, it is difficult to preclude the possible effect of repeat sampling. Secondly, due to the retrospective design of Part 2 of the study, the sample size is limited, and the study is lacking randomization. A prospective randomized controlled trial would allow for more accurate comparison and clarify its clinical value. Our study was conducted quite early between 2017 and 2019, and we have recently revised the rsERT model. It can now predict a precise time window based on a single endometrial biopsy and shows potential clinical value. Clinical trials including multi-center studies are being conducted for validation. Thirdly, a further comparison is required between transcriptomic tools, such as rsERT and ERA.

## Conclusion

In conclusion, our study demonstrated that the novel transcriptomic tool rsERT is superior to the standard marker pinopode in diagnosing WOI and instructing personalized embryo transfer. However, considering the limited sample size, prospective clinical trials and basic research are needed for further validation and clinical application.

## Data availability statement

The original contributions presented in the study are included in the article/**Supplementary Material**, further inquiries can be directed to the corresponding authors.

## Ethics statement

The studies involving human participants were reviewed and approved by the ethics committee of the department of reproductive medicine, Xiangya Hospital, Central South University. The patients/participants provided their written informed consent to participate in this study.

## Author contributions

JC contributed to data analysis and drafted the article. AH and QZ participated in data acquisition. JZ and JF participated in sample collection. HL and YL are the corresponding author, they contributed to the design of the work. All the authors agreed with their contributions and approved the submitted version.

## Funding

This study was supported by the National Key Research and Development Program of China (2021YFC2700404).

## Conflict of interest

The authors declare that the research was conducted in the absence of any commercial or financial relationships that could be construed as a potential conflict of interest.

## Publisher’s note

All claims expressed in this article are solely those of the authors and do not necessarily represent those of their affiliated organizations, or those of the publisher, the editors and the reviewers. Any product that may be evaluated in this article, or claim that may be made by its manufacturer, is not guaranteed or endorsed by the publisher.
