# Supplementary material for: The RNA-seq based endometrial receptivity test (rsERT) compared to pinopode: A better diagnostic tool for endometrial receptivity for patients with recurrent implantation failure in Chinese population
Source: Front Endocrinol (Lausanne). 2022 Oct 21;13:1009161. doi: 10.3389/fendo.2022.1009161 (PMC9634259; doi:10.3389/fendo.2022.1009161)
Supplement: Supplementary file 1 [file Table_1.docx]

**Supplementary table 1.** Demographic characteristics of the patients in Part 1 of the study

| **Parameter** | **Value** |
| --- | --- |
| Patients, n | 49 |
| Age, y | 32.4 ± 3.02 |
| BMI, kg/m^2^ | 21.01 ± 2.26 |
| Infertility duration, y | 4 (3-7.85) |
| Infertility type |  |
| Primary infertility | 31 |
| Secondary infertility | 18 |
| Etiology of infertility |  |
| Tubal | 39 |
| Male | 1 |
| Endometriosis | 2 |
| DOR | 5 |
| Others | 2 |
| Baseline FSH, mIU/ml | 7.01 (6.14-8.30) |
| Baseline LH, mIU/ml | 5.18 (3.09-6.35) |
| AMH, ng/ml | 1.99 (1.17-3.99) |
| Transferred cycles, n | 3.00 (2.00-4.00) |

**Supplementary table 2.** The WOI distribution of the 23 pregnant patients

|  |  | **Pinopode** | | | |
| --- | --- | --- | --- | --- | --- |
|  |  | **Advanced** | **Normal** | **Delayed** | **Total** |
| **rsERT** | **Advanced** | 1 | 0 | 5 | 6 |
|  | **Normal** | 0 | 6 | 9 | 15 |
|  | **Delayed** | 0 | 1 | 1 | 2 |
|  | **Total** | 1 | 7 | 15 | 23 |

**Notes:** The WOI distribution of the 23 patients who conceived successfully after rsERT-instructed pET.

**Supplementary table 3.** Information of the patients with pinopode-instructed pET history

| **Age**  **(y)** | **Failed cycles before pinopode test(n)** | **Pinopode-pET cycles(n)** | **No. of good-quality embryos transferred(n) with pinopode** | **Pinopode*** | **rsERT** | **No. of good-quality embryos transferred(n) with rsERT** | **rsERT-pET pregnancy outcome**** |
| --- | --- | --- | --- | --- | --- | --- | --- |
| 30 | 2 | 1 | 1 | D | A | 2 | 0 |
| 27 | 1 | 2 | 3 | D | A | 2 | 0 |
| 32 | 1 | 2 | 3 | N | N | 1 | 2 |
| 31 | 3 | 1 | 2 | D | A | 1 | 1 |
| 32 | 6 | 1 | 2 | D | N | 0 | 1 |
| 30 | 2 | 2 | 4 | D | A | 2 | 1 |
| 32 | 2 | 1 | 2 | D | N | 1 | 1 |

**Notes:** Seven patients with failed pinopode-instructed pET histories were recruited for rsERT test. Six of them received different results. After conducting pET following the instruction of rsERT, 5 patients conceived successfully on the first attempt. *****Predicted optimal WOI by rsERT or pinopode: A = Advanced WOI (P+3/+4 or LH+5/+6), N = Normal WOI (P+5 or LH+7), D = Delayed WOI (P+6/+7 or LH+8/+9). **Pregnancy outcome: 0=non-pregnant; 1= one gestational sac; 2= two gestational sacs.
